# Anti-inflammatory properties of polysaccharides from edible fungi on health-promotion: a review

**DOI:** 10.3389/fphar.2024.1447677

**Published:** 2024-07-26

**Authors:** Zhenhua Yin, Juanjuan Zhang, Jingjing Qin, Lin Guo, Qingfeng Guo, Wenyi Kang, Changyang Ma, Lin Chen

**Affiliations:** ^1^ Henan Comprehensive Utilization of Edible and Medicinal Plant Resources Engineering Technology Research Center, Huanghe Science and Technology College, Zhengzhou, China; ^2^ National R and D Center for Edible Fungus Processing Technology, Henan University, Kaifeng, China; ^3^ Function Food Engineering Technology Research Center, Kaifeng, China

**Keywords:** edible fungi, polysaccharides, anti-inflammatory effect, structureactivity relationship, health-promotion

## Abstract

Edible fungus polysaccharides have garnered significant attention from scholars due to their safety and potential anti-inflammatory activity. However, comprehensive summaries of their anti-inflammatory properties are still rare. This paper provides a detailed overview of the anti-inflammatory effects and mechanisms of these polysaccharides, as well as their impact on inflammation-related diseases. Additionally, the relationship between their structure and anti-inflammatory activity is discussed. It is believed that this review will greatly enhance the understanding of the application of edible fungus polysaccharides in anti-inflammatory treatments, thereby significantly promoting the development and utilization of edible fungi.

## 1 Introduction

Inflammation, as the initial response of the immune system, is a physiological reaction of the body to injury, infection, and stress (Medzhitov, 2008). Generally, inflammation is a natural protective response that plays a central role in the host defense system by secreting nitric oxide (NO) and pro-inflammatory cytokines. However, uncontrolled long-term or chronic inflammation can be detrimental, leading to tissue damage and numerous diseases, including fever, asthma, rheumatoid arthritis, chronic inflammatory bowel diseases, obesity, diabetes, and cancer (Varela et al., 2018; Barbu et al., 2022; Solier et al., 2023). Currently, the treatment of inflammation primarily involves chemical drugs, including non-steroidal and steroidal anti-inflammatory drugs, which are associated with numerous side effects, such as allergies, osteoporosis, hepatotoxicity, and immunosuppression (Wang and Zeng, 2019; Yu et al., 2019).

Given the critical importance of prebiotics in altering the human gut microbiota and improving host health, edible fungi are gaining attention as one of the healthiest low-calorie foods to promote overall wellbeing (Panda et al., 2024). While not widely used as food sources due to their unique and subtle taste, edible fungi are recognized for their potential in preventing or treating inflammation, cancer, diabetes, and other diseases (Chen H. Y. et al., 2023; Shamim et al., 2023). In fact, they have become very popular health foods because of their rich nutritional contents and low calories. To date, a variety of bioactive components have been extracted from edible fungi, including dietary fibers, polysaccharides, sterols, alkaloids, and terpenoids, which exhibit anti-inflammatory, hypoglycemic, immune-enhancing, and other beneficial activities (Du et al., 2018; Chopra et al., 2021; Yin et al., 2021; Mustafa et al., 2022).

As secondary metabolites, polysaccharides are among the most attractive bioactive components extracted from edible fungi. Maity et al. (2021) and Sun et al. (2022) have reviewed the structure, biological activity, and structure-activity relationship of these polysaccharides, highlighting their immunomodulatory, antibacterial, antioxidant, anti-inflammatory, and anti-tumor activities. As bioactive macromolecules, polysaccharides cannot directly enter cells. However, they can recognize pattern recognition receptors, such as β-glucan receptors and toll-like receptors (TLRs), and activate macrophages, which in turn affect the classical MAPK and NF-κ B signaling pathways, regulate the secretion of related factors, and exert anti-inflammatory effects. Additionally, due to the lack of enzymes capable of decomposing polysaccharides, they are generally considered difficult to digest and absorb in the gastrointestinal tract. Their activity primarily manifests through fermentation reactions by intestinal microorganisms.

**FIGURE 1:**
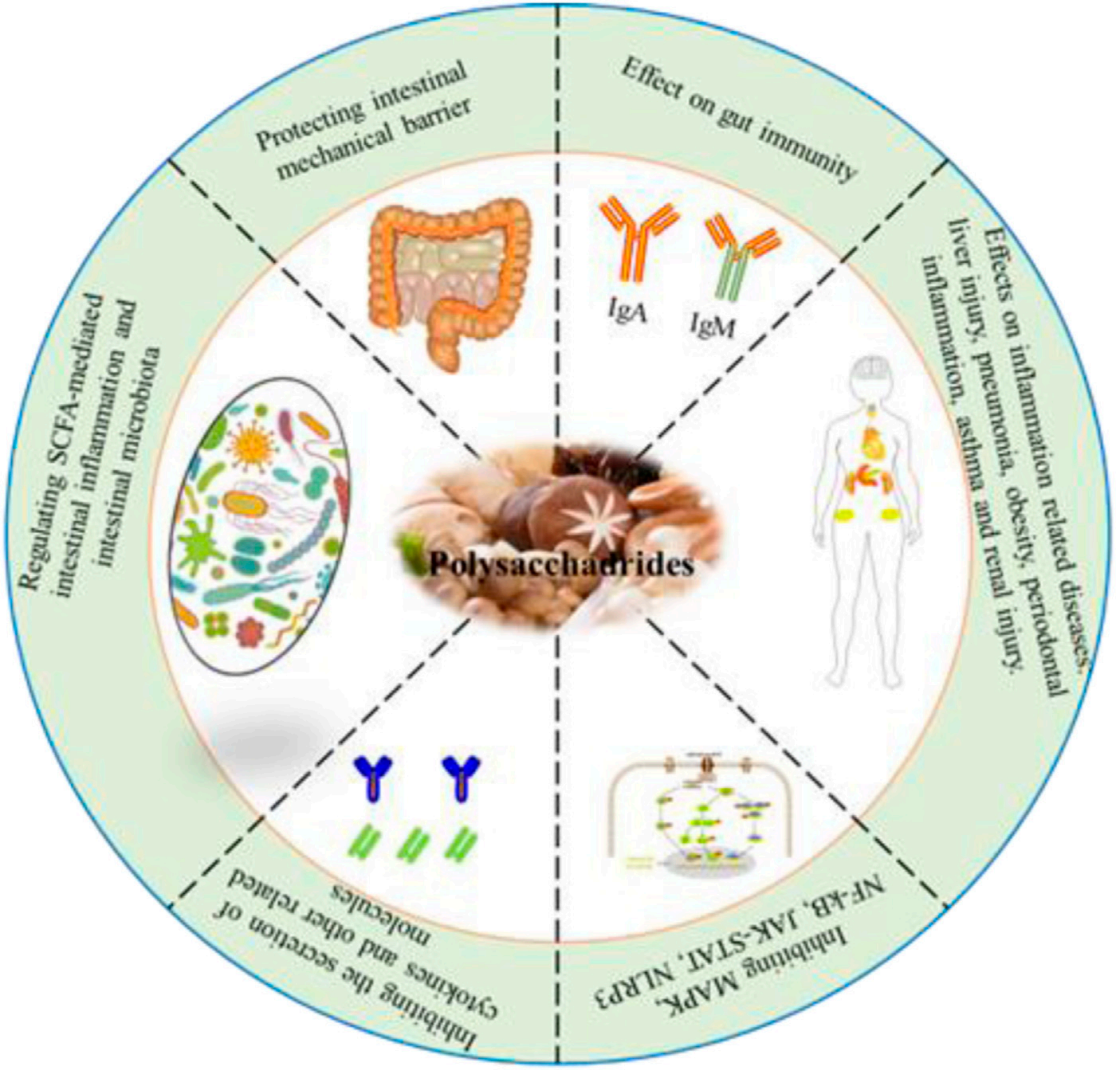
Schematic diagram of anti-inflammatory activity of polysaccharides from edible fungi.

The anti-inflammatory activities of edible fungi polysaccharides have been attracting increasing attention. To date, their potential anti-inflammatory properties have rarely been reviewed. In this work, we provide a comprehensive review of the existing anti-inflammatory activities of edible fungi polysaccharides and analyze their structure-activity relationships to elucidate the potential of edible fungi in the prevention and treatment of inflammation. We believe this review will enhance the understanding of the anti-inflammatory activities of edible fungi polysaccharides and provide valuable guidance for the development and application of new anti-inflammatory drugs. The biological activities of various polysaccharides isolated from edible fungi, such as *Pleurotus ostreatus*, and medicinal edible fungi, such as *Ganoderma lucidum*, are summarized and listed in Figure 1; Supplementary Table S1.

## 2 Anti-inflammation mechanisms of polysaccharides

### 2.1 Effects of polysaccharides on cytokines and other related molecules

Inflammatory cells produce various inflammatory mediators, such as NO, interleukin (IL)-1, IL-6, monocyte chemoattractant protein (MCP)-1 and tumor necrosis factor-*α* (TNF-*α*). TNF-*α* and IL-6 are known to play crucial roles in inflammation, apoptosis, angiogenesis, cell adhesion and transformation (Andaluz et al., 2016). NO is involved in the inflammatory response to tissue injury (Huang et al., 2011). Edible fungi polysaccharides exhibit anti-inflammatory effects by regulating cytokine secretion in inflammatory cells, including those polysaccharides from *Armillaria mellea*, *Cordyceps cicadae*, *Poria cocos*, *G*. *lucidum*, and *Auricularia auricula-judae*. For instance, a xylosyl 1,3-galactofucan (AMPS-III, 500 μg/mL) isolated from *A*. *mellea* significantly suppressed the release of TNF-*α* and MCP-1 in RAW264.7 macrophages and EAhy926 inducted lipopolysaccharide (LPS) and TNF-*α* (Chang et al., 2018). Water-soluble indigestible polysaccharides (NDPs, 40, 80, 160 μg/mL) from *C*. *cicadae* inhibited the secretion of NO, IL-1*β* and TNF-*α* in LPS-stimulated RAW264.7 macrophages (Yang C. H. et al., 2019). An alkali-soluble and carboxymethyl polysaccharide CMP33 from *P*. *cocos* (31.25–1,000 μg/mL) and a water-soluble *β*-1,3-D-glucan with *β*-1,6-D-glucosyl branches polysaccharide GLP-2 (25–100 μg/mL) from *G*. *lucidum* inhibited LPS-stimulated overproduction of NO, IL-6, TNF-*α* and IL-1*β* in RAW264.7 cells (Liu et al., 2019; Jia et al., 2022). A glucuronoxylogalactoglucomannan ME-2 (0.05–1.0 mg/mL) isolated from *A*. *auricula*-judae demonstrated anti-inflammation effects by decreasing the mRNA levels of IL-1*β*, INF-*γ* and TNF-*α* in a dose-dependent manner in LPS-stimulated THP-1 cells (Liang et al., 2023). Additionally, GLP1(1.25 g/L) from *G. lucidum* strain inhibited the expression of IL-1*α* in LPS-induced HaCaT inflammation model (Zhang J. C. et al., 2022). These examples illustrate that polysaccharides from edible fungi exhibit anti-inflammatory activity by regulating the secretion of inflammatory factors.

### 2.2 Effects on inflammatory related signaling pathway

Studies have shown that the secretion of inflammatory cytokines was regulated by mitogen-activated protein kinase (MAPK), nuclear factor kappa B (NF-κB) and Janus kinase-signal transducer and activator of transcription (JAK-STAT) signal pathways. As a key transcription factor, NF-κB signal pathway is associated with pro-inflammatory cytokines and related enzymes, regulating inflammation, immune response, cell division and apoptosis, and playing a crucial role in host defense (Hu et al., 2015; Oh et al., 2019). The MAPK pathway, composed of ERΚ, p38 and JNΚ proteins, is another important signaling pathway that participates in the regulation of inflammatory process through pro-inflammatory mediators and cytokines (Xu J. et al., 2021). JAK-STAT signaling pathway also plays a significant role in regulating the inflammatory response (Schindler et al., 2007). The anti-inflammatory effects of edible fungi polysaccharides are closely related to these signaling pathway. For instance, a proteoglycan LEPS1 (100, 200, 400 μg/mL) from *Lentinus edodes* inhibited the secretion of factors (NO, IL-1*β*, IL-6 and TNF-*α*) by acting on p38 MAPK (p38MAPK) and JAK-STAT1 signaling pathways in LPS-induced RAW264.7 cells (Zhang et al., 2023). WPEP and NPEP from *P. eryngii* significantly inhibited LPS-induced inflammation in RAW264.7 macrophages by regulating the production of NO, prostaglandin E2 (PGE-2), IL-1*β*, TNF-*α* and IL-6, which was related to MAPK and NF-κB signaling pathways (Ma et al., 2020). A exopolysaccharide EPS produced by the medicinal fungus *Cordyceps sinensis* Cs-HK1 significantly inhibited the secretion of NO, TNF-*α* and IL-1*β* in LPS-induced THP-1 and RAW264.7 cells, likely related to the inhibition of NF-kB signaling pathway. EPS also effectively inhibited the expression of TNF-*α*, IL-1*β*, IL-10 and inducible nitric oxide synthase (iNOS) in LPS-induced acute intestinal injury in mice, alleviating intestinal injury (Li L. Q. et al., 2020).

Immunocyte-involved inflammation is thought to regulate the damage associated with various diseases. Oxidative stress, initiated by oxidants such as LPS and reactive oxygen species (ROS), is closely related to chronic inflammation. Additionally, NF-κB signaling pathway and NOD-like receptor thermal protein domain associated protein 3 (NLRP3) inflammasome activation are key mechanisms that regulate the expression of inflammatory cytokines (Du et al., 2019; Hung et al., 2019). A polysaccharide fraction from *Craterellus cornucopioides* (CCPP-1) inhibited LPS-induced accumulation of ROS and NO, reduced the production of TNF-α, IL-1*β* and IL-18, and the expression of iNOS. The mechanism was related to its inhibition of the NF-κB signaling pathway and the activation of NLRP3 inflammasome (Xu J. J. et al., 2021).

**FIGURE 2:**
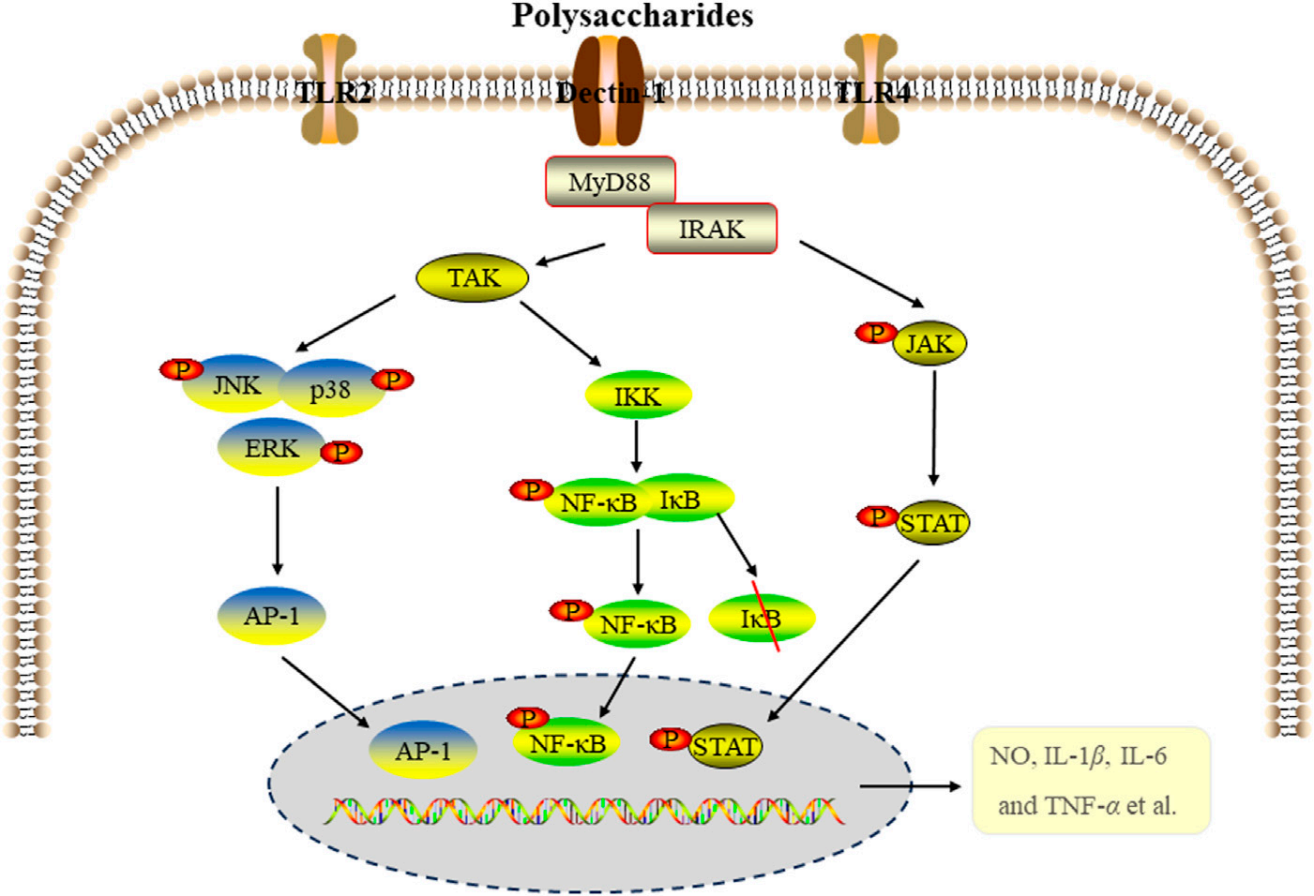
The signal pathways of anti-inflammatory activity of edible fungi polysaccharides.

Polysacchadrides FVP and FFVP from Fermented *Flammulina velutipes* inhibited the secretion and expression of IL-1*β*, IL-6, IL-18 and TNF-*β* in colon tissue, and significantly decreased the expression of NLRP3, ASC, Caspase-1 and IL-1*β* protein in LPS- induced mice model, indicating the anti-inflammatory activity was related to inbibition of the NLRP3 signaling pathway (Ma et al., 2022). Polysaccharide from *P. citrinopileatus* (PCPS) inhibited the secretion of pro-inflammatory cytokines and chemokines by macrophages activated by LPS/INF-γ, and promoted the expression of anti-inflammatory cytokine IL-10. The anti-inflammatory effect was related to Dectin-1 and TLR2 receptors (Minato et al., 2019). There is growing evidence that certain miRNAs play key regulatory roles in macrophage activation and inflammation. miR-155 is closely related to the activation of NF-κB in macrophages, playing an important role in atherosclerosis by inhibiting B cells and promoting the activation of NF-κB in macrophages (Elton et al., 2013; Mann et al., 2017). *Tremella fuciformis* polysaccharides (TFPS) inhibited the inflammatory response of LPS-induced macrophages by inhibiting the expression of miR155 and the activation of NF-κB (Ruan et al., 2018). Figure 2 summarized the MAPK, NF-κB and JAK-STAT signaling pathways involved in the anti-inflammatory activity of edible fungi polysaccharides.

### 2.3 Effects on in inflammatory bowel disease

**FIGURE 3:**
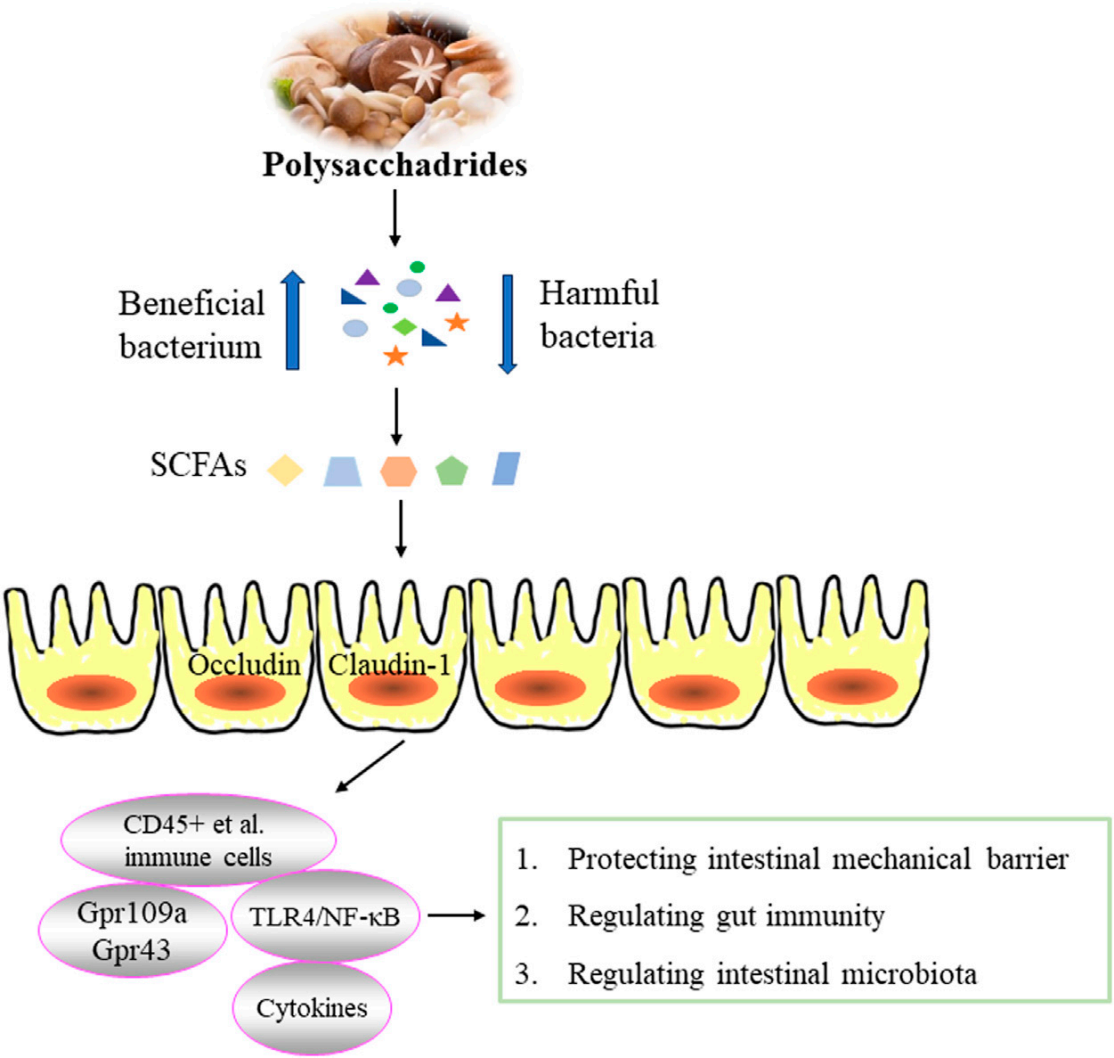
Effect of edible fungi polysaccharides on inflammatory bowel disease.

Inflammatory bowel disease (IBD) is a chronic recurrent inflammatory disease, including crohn disease (CD) and ulcerative colitis (UC). Whlie the pathogenesis of IBD remains unclear, possible pathological mechanisms include immune response disorders, abnormal production of cellular inflammatory factors, impaired intestinal epithelial barrier function and disturbances of intestinal flora (Bisgaard et al., 2022). Edible fungi polysaccharides have been found to relieve intestinal inflammation by regulating the intestinal mucosal barrier. Both intestinal epithelial barrier function and inflammation play crucial roles in the occurrence and development of IBD. The barrier function of intestinal epithelium depends on the integrity of intestinal epithelial cells. Excessive apoptosis of these cells can compromise the barrier, allowing bacteria from the lumen to penetrate the intestinal wall and even bloodstream, potentially leading to septicemia and an inflammatory cascade (Chang, 2020). The intestinal mucosal barrier consists mainly of mechanical, biological, immune and chemical barriers, with the first three barriers being key components of intestinal mucosal immunity. The mechanical barrier is essential for maintaining the integrity of the intestinal mucosal barrier. The intestinal immune barrier involves complex interactions between immune cells and cytokines. The human gastrointestinal tract is colonized by rich and diverse microbial communities that form gut biological barrier and significantly influence the host’s physiology and health (Martínez et al., 2015; Gong et al., 2016). The effect of polysaccharides from edible fungi on inflammatory bowel disease was illustrated in Figure 3.

#### 2.3.1 Effect on intestinal mechanical barrier

Intestinal epithelial cells (IECs) serve as physical and external barriers. Under specific conditions, IECs produce signal molecules such as cytokines to prevent pathogenic microorganisms from entering the intestinal tract. Research has demonstrated that LPS activate Toll-like receptors on IECs, thereby triggering the NF-κB transcription factor pathway, and leading to excessive production of TNF-*α*, IL-6 and IL-8, which can damage IECs (Omonijo et al., 2019; Kayama et al., 2020). Edible fungi polysaccharides exhibit protective effects on intestinal barrier. Polysaccharide from *G. lucidum* spores have been shown to mitigate paclitaxel-induced intestinal barrier injury by reversing microtubule polymerization and inhibiting apoptosis (Li D. et al., 2020). Furthermore, a water-soluble polysaccharide (GLSP) from *G. lucidum* demonstrated potent anti-inflammatory activity by inhibiting the excessive production of NO, IL-6 and IL-1*β* in IEC-6 cells induced by LPS, suggesting the potential of GLSP in protecting the gut barrier (Wen et al., 2022).

#### 2.3.2 Effect on gut immunity

The host immune system also plays a crucial role in the development of IBD. Previous studies have shown that EP-1 effectively alleviates symptoms in acetic acid-induced UC rats by reducing IL-1 and IL-6 levels, increasing superoxide dismutase (SOD) levels and decreasing malondialdehyde (MDA) levels. It also lowers complement 3 (C3) and IgM levels, demonstrating anti-inflammatory, antioxidant and immune-enhancing activities (Shao et al., 2019). WPEP has been found to significantly reverse symptoms of colitis induced by dextran sulfate sodium (DSS) in mice. It reduces the concentration of pro-inflammatory cytokines and the expression of pro-inflammatory proteins, increases colon length, improves histology. These effects are associated with reduced accumulation of CD45^+^ immune cells, CD45+F4/80 + macrophages and CD45^+^ Gr1+ neutrophils (Ma et al., 2021). Studies have proved that Foxp3+T cells can inhibit inflammation and the production of IgA. *T*. *fuciformis* polysaccharides (TPs) have shown a protective effect against DSS-induced colitis in mice by regulating immune system. This effect involves reducing the Foxp3+T cells and IgA-coated bacteria, decreasing pro-inflammatory cytokines, and enhancing anti-inflammatory cytokines (Xu Y. et al., 2021).

#### 2.3.3 Effect on intestinal microbiota-related IBD

Due to the absence of carbohydrate-active enzymes, most polysaccharides cannot be directly digested or absorbed by the body. Instead, their primary activity occurs through the fermentation by intestinal microorganisms (Kong et al., 2016; Ma et al., 2017). Abnormal changes in intestinal microflora can induce inflammation and exacerbate various inflammatory diseases (Schippa and Conte, 2014; Zuo and Ng, 2018). Edible fungi polysaccharides have been shown to regulate intestinal health by modulating intestinal microorganisms. For example, *Scorias spongiosa* polysaccharides SSPs have demonstrated a capacity to decrease the levels of IL-1*β*, IL-6 and TNF-*α,* while increasing IL-10 level to enhance anti-inflammation abilitiy. They also alter microbial community and composition by increasing the abundances of Firmicutes, Campilobacterota, Desulfobacterota, Proteobacteria, Actinobacteria, and Fusobacteria, Bacteroidetes, and Verrucomicrobia, while decreasing Verrucomicrobiota, Bacteroidota, Patescibacteria, and Synergistota in C57BL/6J mice (Xu Y. et al., 2022). Lentinan have been observed to inhibit the expression of cytokines (TGF-*β*, TNF-*α*, IL-1*β*, IL-6 and IL-8), attenuate IκBα degradation in LPS-induced inflammatory response in juvenile taimen intestine. Additionally, Lentinan increase the relative abundance of beneficial bacteria such as Lactobacillaceae, Lachnospiraceae and Ruminococcaceae while reducing harmful bacteria like Enterobacteriaceae, Fusobacteriaceae and Flavobacteriaceae. These effects suggest that the anti-inflammatory properties may correlate with NF-κB signaling pathway and improvement of intestinal microflora (Ren et al., 2019). The inhibitory effect of WPEP on DSS-induced colitis in mice is closely associated with intestinal microflora imbalance. It partially reverses this imbalance by decreasing the abundance of *Ackermanella myxophilus* and *Clostridium cocleatum*, while increasing *Bifidobacterium pseudocolon*, *Lactobacillus*, *Lactobacillus saliva* and *Bromotococcus* abundance (Ma et al., 2021). Similarly, TPS has shown significant ability to increase intestinal community diversity and restore the relative abundance of *Lactobacillus*, *Odoribacter*, *Helicobacter,* Ruminococcaceae, and Marinifilaceae in DSS-induced colitis in mice (Xu Y. et al., 2021).

Polysaccharides serve as an energy source for intestinal microorganisms, promote their proliferating and the production of beneficial compounds. The metabolization of polysaccharides by intestinal flora generates short-chain fatty acids (SCFAs), which possess immunomodulatory and anti-inflammatory activities critical for maintaining intestinal homeostasis, regulating immune function, and mitigating intestinal inflammation (Venegas et al., 2019). SCFAs help maintain a low pH environment that inhibits pathogen growth and stimulates the growth of butyric acid-producing bacteria, thereby reinforcing intestinal immune barriers (Gonçalves and Martel, 2016). For example, *G*. *lucidum* polysaccharide (GLP) have shown potential in alleviating DSS-induced colitis in mice by increasing SCFAs-producing bacteria, reducing pathogens in the small intestine and cecum, and enhancing SCFAs production (acetic acid, propionic acid and butyric acid). GLP also regulated the expression of genes involved in six inflammation-related KEGG pathways, thereby enhancing immunity, reducing inflammatory response and potentially lowering the risk of colon cancer (Xie et al., 2019). Similarly, the starch-free *β*-type glycosidic polysaccharide FVP from *F*. *velutipes* exerted protective effects in DSS-induced UC mice. FVP regulated the relative mRNA expression of cytokines (TNF-*α*, IFN-*γ*, IL-1*β*, IL-6, MCP-1 and MIP-1*α*) and tight junction proteins (caludin-1, occluding and zonulae occludens-1), modified intestinal microflora, and increased levels of acetic acid, propionic acid and n-butyric acid (Zhao et al., 2020). Administration of FVP (50 mg/kg and 100 mg/kg) reduced inflammatory response in DSS-induced colitis, significantly inhibited myeloperoxidase (MPO) activity, decreased levels of DAO and NO, and effectively restored the metabolic balance of intestinal microorganisms, especially promoting butyric acid production. These actions contribute to down-regulating the Toll-like receptor 4 (TLR4)/NF-κB inflammatory signaling pathway, thus improving colitis symptoms (Zhang et al., 2020).

SCFAs exert their immunomodulatory and anti-inflammatory effects by inhibiting histone deacetylases and activating G protein-coupled receptors (Gpr) such as Gpr43 and Gpr109a on the surface of intestinal epithelial cells and immune cells (Kim, 2018). These actions are crucial for maintaining intestinal homeostasis and controlling inflammation. For example, EP-1 has been shown to effectively alleviate acetic acid-induced UC in rats by regulating intestinal microflora. EP-1 also increased the relative proportions of acetic acid and butyric acid in feces and inhibited the expression of Gpr41 and Gpr43 (Shao et al., 2019). Similarly, the heteropolysaccharide FVP from *F*. *velutipes* demonstrated anti-inflammatory properties by inhibiting intestinal inflammation, regulating intestinal permeability, and reducing intestinal injury. FVP also prevented the downregulation of tight junction genes (Occludin and Claudin-1) and Gpr43 and Gpr109a induced by Cd. Moreover, PVP altered intestinal flora composition, and enhanced SCFAs production. These mechanisms collectively contributed to FVP’s ability to mitigate CdCl_2_-induced intestinal toxicity and damage by modulating SCFA-mediated intestinal inflammation and energy metabolism related to intestinal microbiota (Hao et al., 2023).

#### 2.3.4 Other effect on IBD

A caspase-independent form programmed cell death known as necroptosis has been identified as playing a significant role in in the pathogenesis of IBD (Rosenbaum et al., 2010). Polysaccharides extracted from *L. edodes* have shown dose-dependent inhibition of DSS-induced colitis in mice and have been observed to suppress necrotic cell death in Caco-2 cells. Notably, these polysaccharides exerted pronounced inhibitory effect on the necroptosis signaling cascade involving receptor-interacting protein kinase receptor-interacting protein kinase 1/receptor-interacting protein kinase 3/mixed kinase-region-like proteins (RIPK1-RIPK3-MLKL), resulting in decreased levels of phosphorylated MLKL in colitis mice. This inhibition of necroptotic cell death in the colon might contribute to the anti-inflammatory effects of *L. edodes* polysaccharides (Alagbaos and Mizuno, 2021). Furthermore, *L*. *edodes* polysaccharides have been shown to mitigate weight loss and reduce the expression of pro-inflammatory cytokines such as TNF-*α*, IL-6, IL-1*β* and IFN-*γ* in DSS-induced colitis in mice, suggesting their potential therapeutic efficacy in colitis treatment. Additionlly, evidence indicated that necroptosis might be linked to the expression of pro-inflammatory cytokines, further underscoring the anti-inflammatory mechanisms (Alagbaoso and Mizuno, 2022).

### 2.4 Effects on inflammation related diseases

#### 2.4.1 Effect on inflammation of liver injury

The Liver, a crucial organ in the human body, is susceptible to damage from toxic substances and drugs (Asrani et al., 2019). In recent years, chemical-induced liver injury has become increasingly significant in term of its impact on human health (Meng et al., 2018b). Studies have consistently shown that acute liver injury is often involved excessive oxidative stress and inflammatory responses (Dai et al., 2021; Yan et al., 2023). Tetrachloromethane (CCl_4_) is well-known environmental toxin widely used to induce experimental liver damage due to its ability to induce oxidative stress and inflammation (Liu et al., 2017; Wang et al., 2022). Edible fungi polysaccharides have demonstrated protective effects against CCl_4_-induced liver injury. For example, *Morchella importuna* polysaccharides (Mw 35.54 kDa) and *F. velutipes* polysaccharides (FVPs) have shown to mitigate CCl_4_-induced liver injury by enhancing antioxidant defenses and reducing inflammatory (Xu et al., 2021; Xu Y. et al., 2022; Xu et al., 2022 Y. Y.). *P. ostreatus* polysaccharide POP has exhibited hepatoprotective effects against CCl_4_-induced acute lung injury (ALI), attributed to its antioxidant properties that regulate metabolic pathway disorders and mitigate liver mitochondrial apoptosis (Zhu et al., 2019). Additionally, phosphorylated POP (PPOP) has shown stronger protective effects compared to POP, possibly through scavenging free radicals, preventing lipid peroxidation, and enhancing the endogenous antioxidant defense system (Duan et al., 2020). Abnormal activation of the NLRP3 inflammasome, leading to the release of pro-inflammatory cytokines, played a significant role in various types of ALI (Shao et al., 2020; Yu et al., 2023). *G. lucidum* polysaccharides (GLPS) have also demonstrated anti-inflammatory and hepatoprotective effects against CCl_4_-induced liver injury by inhibiting the inhibiting of NLRP3 activation, reducing inflammation, and inhibiting lipid peroxidation induced by free radicals (Chen et al., 2019).

Certainly, various factors such as alcohol, DSS and LPS can cause liver injury, with oxidative stress and inflammation being pathological mechanisms. For example, a heteropolysaccharide (EPS) from *P. geesteranus* has been shown to protect against alcohol-induced liver injury. The protective effect is likely achieved through enhancing antioxidant defenses and reducing anti-inflammatoion (Song et al., 2018). Similarly, a hepatoprotective polysaccharide PSP-1b1 (80 and 160 mg/kg/day) from *Coriolus versicolor* demonstrated protective effects against alcohol-induced liver injury by mitigating oxidative stress and modulating immune responses (Wang et al., 2019). *M. esculenta* polysaccharides have also bene studied for their protective effects against DSS-induced liver injury, primarily attributed to their ability to reduce oxidative stress, inhibit inflammation and enhance the activity of liver antioxidant enzymes (Chen S. T. et al., 2023). Additionally, GFP has shown efficacy in reducing liver injury induced by LPS/D-galactosamine (D-GalN) in mice. Its protective mechanism involved antioxidant defense and anti-inflammatory effects, likely mediated through inhibition of the miR-122/nuclear factor erythroid 2-related factor 2 (Nrf2)/anti-oxidative response element (ARE) pathway (Meng et al., 2021).

**FIGURE 4:**
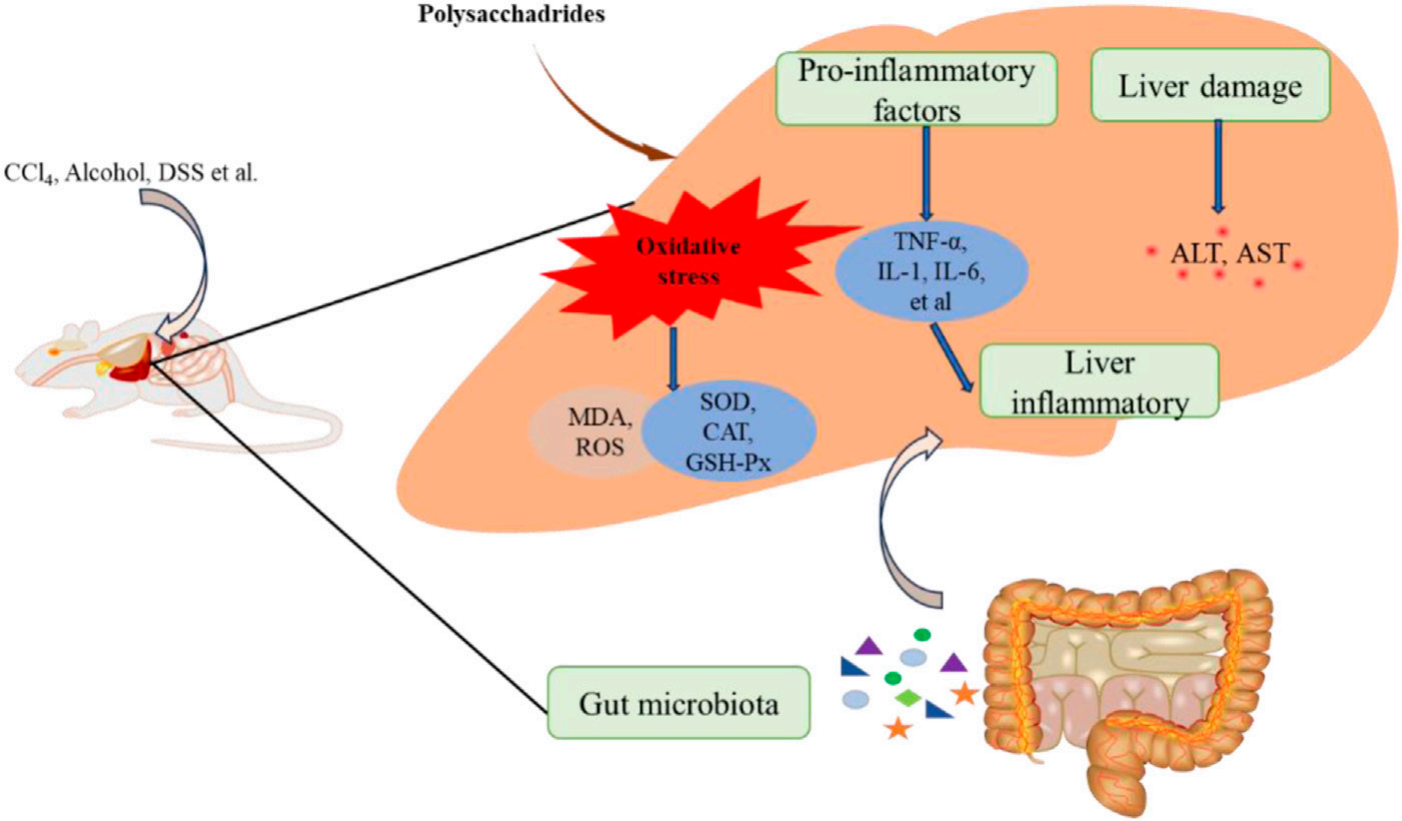
The hepatoprotective effect of edible fungi polysaccharides.

Dysregulation of gut microbes can compromise the integrity of intestinal barrier, allowing translocated bacteria and intestinal by products to enter the liver through the portal vein. This process increases oxidative stress and inflammation in the liver, posing a threat to live health and function. Increasing evidence suggests that gut microbes play a crucial role in protective effects of natural products on liver health (Meng et al., 2018a). For example, FVPs has been shown to alter the composition of intestinal microflora. It regulated bacterial pathways involved in fatty acid biosynthesis, tryptophan metabolism and exogenous metabolism via cytochrome P450, thereby protecting the liver from the toxic effects of CCl_4_ (Xu Y. Y. et al., 2022). Similarly, *Coprinus comatus* polysaccharide (CCP) has demonstrated the ability to modify the structure of intestinal flora. It inhibited the proliferation of *Clostridium perfringens*, Enterobacteriaceae and *Enterococcus*, while promoting the growth of *Lactobacillus* and *Bifidobacterium* in the gut of ALD mice. This modulation contributed to its anti-alcoholic liver injury effects (Yu et al., 2024). The relevant hepatoprotective effect was shown in Figure 4.

#### 2.4.2 Effect on inflammation of pneumonia

Since the outbreak of COVID-19 in 2019, there have been more than 500 million clinically confirmed cases worldwide. The outbreak of COVID-19 has led to significant increase in the number of acute pneumonia cases (Song P. P. et al., 2020). Acute pneumonia is a respiratory disease characterized by diffuse inflammatory lung injury, which can be caused by lung tissue contusion, bacterial, or virus infections. Activation of immune cells and excessive release of pro-inflammatory mediators are fundamental aspects of its pathogenesis (Bakowitz et al., 2012; He et al., 2021). LPS, as an endotoxin, is used to induce acute lung injury, triggering robust inflammation and immune response. Polysaccharides of edible fungi have shown protective effects against LPS-induced lung injury. For instance, *G. lucidum* polysaccharides (GLP, 25, 50 and 100 mg/kg/d) inhibited inflammatory cells infiltration, reduced the release of granulocyte macrophage-colony stimulating factor (GM-CSF) and IL-6, and decreased gene expression of IL-1*β*, IL-6, TNF-*α* and serum amyloid A3 (Saa3). Additionally, GLP inhibited neuropilin-1 (Nrp1) activation, upregulated B-cell lymphoma-2/Bcl-2-associated X protein (Bcl2/Bax) and Lc3 levels, and downregulated C-Caspase3/Caspase3 and p62 expression. These actions suggested that GLP protected against pneumonia by blocking inflammatory cells infiltration, suppressing cytokines secretion, inhibiting Nrp1 activation, regulating alveolar cell apoptosis, and modulating autophagy (Zhang X. L. et al., 2022). Residue polysaccharides (RPS) from *L*. *edodes* residue powder and its hydrolysates, acidic-RPS (ARPS) and enzymatic-RPS (ERPS), also showed lung protective effects in LPS-induced lung injured in mice. ERPS showed superior effects by reducing lung wet-to-dry weight ratio, inflammatory factors (TNF-*α*, IL-6, and IL-1*β*), complement C3 (C3) and high-sensitivity C-reactive protein (hs-CRP), while mitigating oxidative stress (Ren et al., 2018). *L*. *edodes* polysaccharides (PLE), primarily composed of Glc, Gal, GlcA, and Man, protected lung tissue by reducing hs-CRP and C3 levels, inhibiting gamma-glutamyltransferase (GGT) activity, decreasing TNF-*α*, IL-1*β* and IL-6 levels, and enhancing antioxidant enzymes SOD and catalase (CAT) activities (Zhang Y. W. et al., 2022).

**FIGURE 5:**
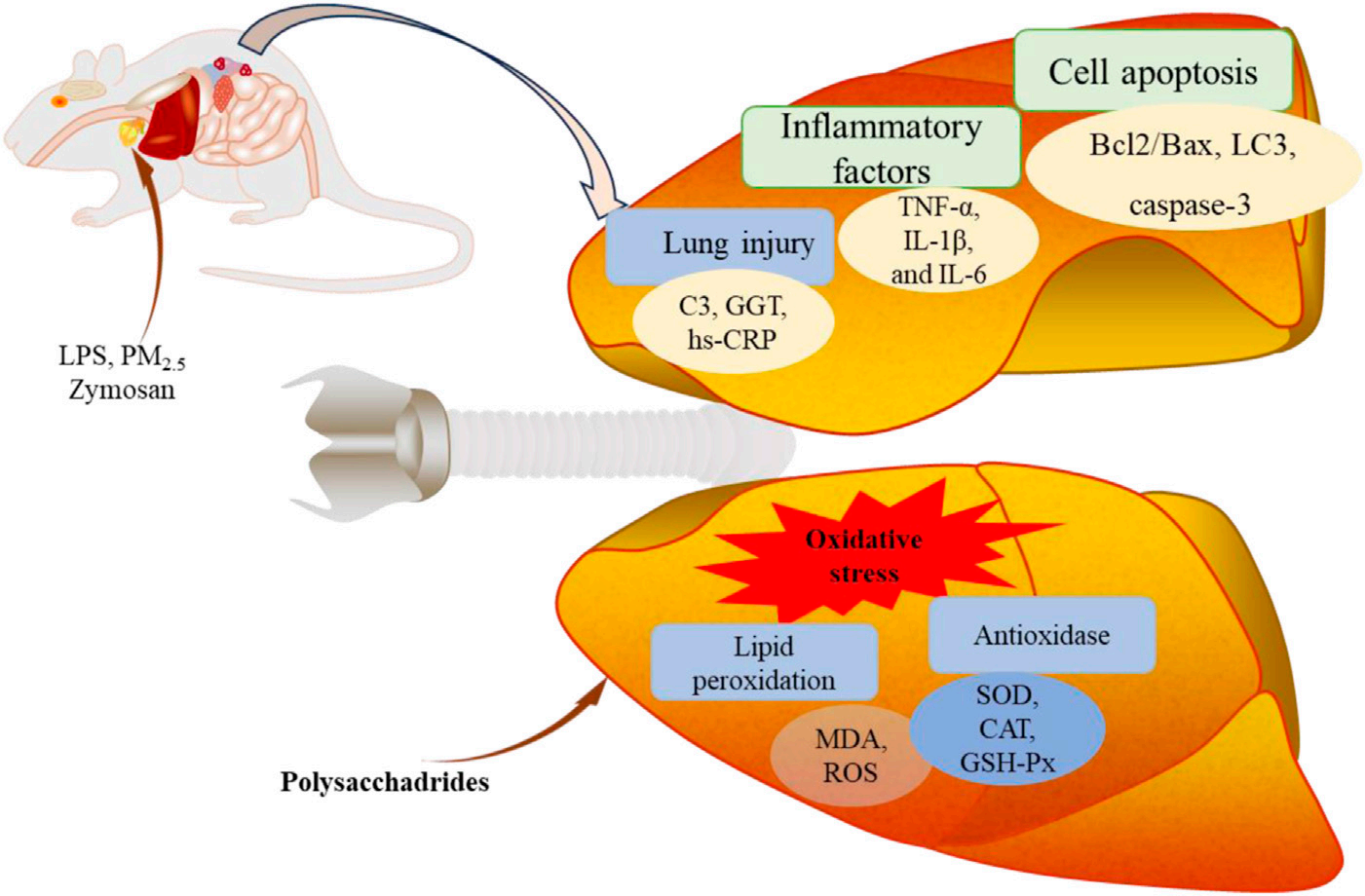
The protective effect of edible fungi polysaccharides on lung injury.

Inhalation of fine particulate matter (PM_2.5_) can induce systemic inflammation, thereby increasing the risk of lung injury (Yan et al., 2017). Alveolar macrophages, upon encountering PM_2.5_ particles in the lungs, become activated and release cytokines and chemokines that recruit inflammatory cells to the lung, leading to inflammation (Bekki et al., 2016). NF-κB is a key transcription factor dimer that regulates the expression of pro-inflammatory cytokines such as TNF-*α* and IL-1*β*, and it is central to the pathogenesis of PM_2.5_-induced lung disease (Li et al., 2018). Sulfated polysaccharide from *M*. *esculenta* (SFMP-1) has shown protective effects against cell death, apoptosis, production of TNF-*α* and IL-1*β* in rat alveolar macrophages (NR8383 cells) induced by PM_2.5_, Its mechanism was involved inhibition of NF-κB activation (Li et al., 2019). PM2.5 exposure also induces antioxidant damage. *Trametes orientalis* polysaccharide (TOP-2) attenuated PM_2.5_-induced lung injury in mice through its antioxidant and anti-inflammatory effects, partly mediated by activating Nrf2/Heme oxygenase-1 (HO-1) pathway and inhibiting NLRP3 inflammasome (Zheng et al., 2019). The relevant protective effect was shown in Figure 5.

#### 2.4.3 Effect on inflammation of obesity

Obesity is characterized by chronic low-grade inflammation, and the relationship between chronic inflammation and obesity has been extensively studied. Two polysaccharides (CPA-1 and CPB-2) isolated from *C*. *cicadae* have been found to possess protective effects on HFD/HF-induced insulin resistance, metabolic abnormalities, hepatic oxidative stress (MDA, GSH-Px, SOD and CAT) and inflammatory response (TNF-*α*、IL-1β and IL-6) (Zhang et al., 2021). In mice fed a high-fat diet, water-soluble glucan from *Grifola frondosa* (GFPA) has demonstrated the ability to mitigate liver steatosis and inflammation, along with promoting significant weight loss. These beneficial effects were attributed its inhibition of chronic inflammation through the TLR4/NF-kB signaling pathway (Jiang et al., 2022).

Intestinal microflora plays a crucial role in the onset and progression of obesity by influencing host energy metabolism, substrate metabolism, and inflammation. *Dictyophora indusiata* polysaccharides have been shown to exert anti-obesogenic and anti-inflammatory effects by modulating the intestinal microbiome and inflammatory pathways in mice HFD-fed diet (Kanwal et al., 2020). Polysaccharide extracted from the sporoderm-broken spores of *G*. *lucidum* (BSGLP) has demonstrated significant reduction in fat accumulation, liver steatosis, inflammation, and hyperlipidemia in high-fat diet-fed mice. Its effects were believed to involve regulation of the intestinal microflora, enhancement of intestinal barrier function, promotion of SCFAs production, activation of GPR43 and inhibition of TLR4/myeloid differentiation factor 88 (MYD88)/NF-κB signaling pathway (Sang et al., 2021). Polysaccharide from *Agrocybe cylindracea* (ACP) has been found to ameliorate obesity in high-fat diet-induced obese mice by significantly reducing the levels of obesity-related TNF-*α* and IL-6. This effect partly results from decreasing the abundance of *Desulfovibrio* and increasing the abundance of *Parabacteroides*, along with related changes in *solaventivone* levels (Zhu et al., 2022).

#### 2.4.4 Effects of inflammation on other diseases

Edible fungi polysaccharides have demonstrated potential in inhibiting periodontal inflammation, asthma, and providing renal protection. For instance, crude polysaccharides (CGLPs) from *G*. *lucidum* sourced from Changbai Mountain were found to regulate the expression of IL-1*β*, TNF-*α* and IL-10 in a concentration-dependent manner, effectively inhibiting alveolar bone loss caused by periodontitis (Chen Z. et al., 2023). In the context of allergic asthma, *C. militaris* polysaccharide CMP has been shown by Song et al. to mitigate oxidative stress and inflammation in mice with allergic asthma. The effect was achieved through the activating of NRF2/HO-1 signaling pathway and inhibiting of NF-κB signaling pathway. Importantly, these mechanisms were closely linked to maintaining the stability of intestinal microflora, highlighting the multifaceted protective roles of polysaccharides from edible fungi (Song et al., 2023).

ASMCP extracted from spent mushroom compost of *L*. *edodes* has been shown to decrease the levels of TNF-*α*, IL-6 and IL-1*β*, demonstrating potential anti-inflammatory activity on LPS-induced renal injury in mice (Song et al., 2020b). *C*. *cicadae* polysaccharide CCP has been reported to alleviate renal injury and tubulointerstitial fibrosis in rats with high-fat diet and STZ-induced diabetic nephropathy. This effect was achieved by CCP inhibiting the TLR4/NF-κB and TGF-*β*1/Smad signaling pathways, thereby reducing inflammatory reactions and modulating intestinal microflora (Yang et al., 2020). Additionally, crude polysaccharides from *Floccularia luteovirens* (FLPs) have shown effectiveness in improving renal tissue injury induced by high glucose. They targeted and regulated phosphorylated glycogen synthase kinase3*β* (GSK-3*β*), and inhibited the accumulation of inflammatory factors, highlighting their potential in renal protection (Wang et al., 2023).

## 3 Structure-anti-inflammatory activity relationship

The biological activities of polysaccharides are decisively influenced by their structures and physicochemical properties. Key factors include molecular weight, monosaccharide composition, type of linkage, degree of branching, conformation and solubility, all of which contribute to their anti-inflammatory activities. These structural attributes determine how polysaccharides interact with biological systems, influencing their efficacy in modulating immune responses and inflammatory processes. Understanding these structural features are crucial for elucidating and optimizing the therapeutic potential of polysaccharides in various health applications.

Research indicates that the specific glucans’ recognition and binding by immune cells can lead to subsequent immunomodulatory and anti-inflammatory activities (Camilli et al., 2018; Nakashima et al., 2018). Factors such as molecular weight and connection mode of polysaccharides are crucial in determining their receptor binding properties and anti-inflammatory activities. For example, the high molecular weight fraction of *G. frondosa* (GF70-F1, 1,260 kDa) has been shown to inhibit TNF-*α* and IL-6 production, along with NF-κB activation in LPS-induced RAW264.7 cells. This activity was likely mediated through interaction with TLR2 receptors rather than Dectin-1 or CR3 receptors due to its (1→6)-branched (1→4)-*β*-D-glucan structure (Su et al., 2020). In another study, polysaccharides (wHEP-1, wHEP-2 and wHEP-3) isolated from the mycelium of *Hericium erinaceus* demonstrated varied anti-inflammatory activities, with the high molecular weight wHEP-1 exhibiting the most potent effects in LPS-induced Caco-2 cells and a rat model (Wang et al., 2021). Similarly, *β-*Glucan H6PC20 (2,390 kDa) and *α*-heteropolysaccharide HPB-3 (15 kDa) isolated from *H*. *erinaceus* showed protective effects against alcoholic gastric ulcer in rats, with HPB-3 focusing specifically on anti-inflammation actions (Chen et al., 2020). Furthermore, Ma et al. (2020) have highlighted that low molecular weight FFVP (15,702 Da) exhibited was higher anti-inflammatory ability than that of high molecular weight FVP (15,961 Da). These findings underscore the importance of polysaccharide structural characteristics in influencing their biological activities, particularly in modulating inflammation through various receptor interactions.

The conformations of polysaccharides play significant roles in their anti-inflammatory activities. Yang et al., confirmed this by studying water-soluble indigestible polysaccharide NDPs (24.4 kDa) from *C*. *cicadae*. They found that NDPs laking a triple helix conformation strongly inhibited the production of NO, IL-1*β* and TNF-*α* by LPS-stimulated RAW264.7 macrophages compared to the crude polysaccharide CP (3.1 kDa, 21.5 kDa, 678.2 kDa) that possessed a triple helix conformation (Yang C. H. et al., 2019). This suggests that the absence of a triple helix conformation in NDPs enhances their anti-inflammatory effectiveness, highlighting the importance of polysaccharide conformation in influencing their biological activities.

The monosaccharide composition of polysaccharides indeed plays a significant role in their anti-inflammatory activities. For example, in the case of polysaccharides derived from the residue of *L*. *edodes* (RPS, ARPS, and ERPS), they have shown notable pulmonary protective effects. Among these, ERPS demonstrated superior efficacy, with Rha presumed to be essential for its lung protective activity (Ren et al., 2018). This highlights the importance of specific monosaccharide compositions in influencing the biological properties and therapeutic potential of polysaccharides.

Chemical modification and the addition of new chemical groups can indeed enhance the activities of polysaccharides or impart them with new therapeutic properties. For example, in studies on polysaccharides from *M. angusticeps* Peck, phosphorylation (PMEP) and three acetylated polysaccharides Ac-PMEP_1-3_ were employed to modify their structures. Among these, Ac-PMEP3, with a highly branched structure, demonstrated stronger anti-inflammatory effects by inhibiting the excessive production of NO and TNF-*α* in LPS-induced RAW264.7 cells (Yang Y. X. et al., 2019). Similarly, acetylated polysaccharide AcPPS from *P*. *ostreatus* exhibited lung protective effects in zymosan-induced acute lung injury mice, with the mechanism linked to the NF-κB signaling pathway (Song et al., 2020c). Phosphorylated polysaccharides PMPS from *P. djamor* mycelia showed antioxidant, anti-inflammatory, and anti-fibrotic effects in adenine-induced chronic renal failure (CRF) mice, highlighting phosphorylation as an effective modification method (Li et al., 2021). Additionally, sulfated polysaccharide from *L*. *edodes* (SPLE), characterized by sulfation of its *β*-glucan structure, demonstrated anti-inflammatory effects in zymosan-induced multiple organ dysfunction syndrome in mice (Sun et al., 2021). These examples underscore how chemical modifications can enhance or alter the biological activities of polysaccharides, expanding their therapeutic potential in various disease contexts.

## 4 Conclusion and further perspective

Polysaccharides derived from edible fungi have garnered significant scholarly interest due to their safety profile and promising biological activities. This paper comprehensively reviews their anti-inflammatory activities, mechanism of action, and the effects on related inflammatory diseases. Special emphasis is placed on exploring the relationship between polysaccharide structure and anti-inflammatory activity. By synthesizing current knowledge, this review aims to deepen our understanding of how edible fungi polysaccharides can be applied in inflammation-related contexts. Ultimately, this research is expected to catalyze advancements in the development and utilization of edible fungi for therapeutic purposes.

Edible fungi polysaccharides exert their anti-inflammatory effects by inhibiting the release of relevant factors through interactions with membrane receptors and suppression of specific signaling pathways. Additionally, they can mitigate inflammation by enhancing intestinal mechanical and intestinal immune barriers, as well as modulating intestinal microorganisms. These polysaccharides have shown potential in addressing inflammation-related conditions such as liver injury, obesity, asthma, glomerulonephritis, and periodontitis. However, most research on their anti-inflammatory activities has been conducted in cell cultures or animal models, which may not fully reflect their effects in humans. Therefore, further clinical studies are needed to validate their therapeutic potential in human applications. The precise mechanisms underlying the anti-inflammatory actions of edible fungi polysaccharides remain incompletely understood. It is anticipated that future research will uncover additional signaling pathways and molecular targets relevant to inflammation. Structure-activity relationship analyses indicate that the structural characteristics of polysaccharides significantly influence their anti-inflammatory activities. Additionally, anti-inflammatory activities of polysaccharides also depend on the degradation, absorption and utilization processes. The complex structure of polysaccharides allows them to evade the action of human digestive enzymes, thereby forming a specific digestive pattern. Therefore, understanding the metabolic process of polysaccharides is of great significance for exploring the benefits and scientific applications of polysaccharides on host health. However, the complexity of polysaccharide structures presents challenges in fully elucidating these relationships. Therefore, there is a critical need for continued investigation into the structure-function dynamics of polysaccharides, with a focus on structural modifications to optimize their therapeutic efficacy. This approach holds promise for maximizing the beneficial effects of edible fungi polysaccharides in combating inflammation and advancing their clinical applications.
