# Supplementary material for: Anti-inflammatory properties of polysaccharides from edible fungi on health-promotion: a review
Source: Front Pharmacol. 2024 Jul 26;15:1447677. doi: 10.3389/fphar.2024.1447677 (PMC11310034; doi:10.3389/fphar.2024.1447677)
Supplement: Supplementary file 1 [file Table1.pdf]

Table S1 Summary of source, extraction, purification, structure and anti-inflammatory activity of polysaccharides from Edible Fungi

No	Source	Name	Extraction and purification methods	Mw (kDa)/ detection method	Monosaccharide composition/detection method	Experimental model	Anti-inflammatory activity	Reference
1	Mycelium of *Armillaria mellea*	AMPS-III	Fractionated by size-exclusion column chromatography	13/HPSEC	Fuc, Gal, and Xyl in a ratio of 6:5:4/HPLC	LPS and TNF-*α* induced RAW264.7 macrophages and EAhy926	Suppressing the release of TNF-*α* and MCP-1	Chang et al. (2018)
2	Fruiting bodies of *Cordyceps cicadae*	NDPs	Enzymolysis, dialysis, deproteinization, alcohol deposition	24.4/HPGFC	Man (50.4%), Gal (33.4%), Glc (13.7%), Ara (1.4%) and Rib (1.0%)/HPLC	LPS induced RAW264.7 macrophages	Inhibiting the secretion of NO, IL-1*β* and TNF-*α*	Yang et al. (2019a)
3	*Poria cocos*	CMP33	Alkaline extraction followed by DEAE-52 and Saphadex-G200 and Saphadex-G150 column chromatographies.	152.3/HPGPC	Glc/GC–MS	LPS-induced RAW264.7 cells	Inhibiting the production of NO, IL-6, TNF-*α* and IL-1*β*	Liu et al. (2019)
4	Fruiting bodies of *Ganoderma lucidum*	GLP-2	Microwave-assisted freeze-thaw extraction, purification with Sepharose CL-6B chromatography	167/SEC-RI-MALLS	Glc/GC	LPS-induced RAW264.7 macrophages	Inhibiting the production of NO, TNF-*α*, IL-1*β*, and IL-6	Jia et al. (2022)
5	Fruiting bodies of *Auricularia auricula-judae*	ME-2	Isolation by FPA90-Cl and DEAE 650 M	260/SEC-MALLS-VIS-RID	Xyl, GlcA, Glc, Man and Gal in an experimental molar percentage of 23.0 %, 4.3 %, 5.2 %, 64.3 % and 2.7 %/RPLC-QQQ-MS	LPS-stimulated THP-1 cells	Decreasing the mRNA levels of IL-1*β*, INF-*γ*, and TNF-*α* in a dose-dependent manner.	Liang et al. (2023)
6	Mycelium of *Ganoderma lucidum*	GLP1	Extraction with water at a ratio of 1:30 (m/V) for 1 h at 70°C, separation with DEAE-52 column chromatography	37.5 and 18.3/HPLC	-	LPS-induced HaCaT inflammation model	Inhibiting the expression of IL-1*α*	Zhang et al. (2022a)
7	Dry spent substrate of *Lentinula edodes*	LEPS1	Enzymolysis, isolation with Sephacryl S-300 HR column	1.18×10^4 g/mol/HPSEC	Glc, Ara, Gal, Xyl and Man at a molar ratio of 1.2: 1.2: 1.0: 2.3: 1.1/HPAEC	LPS-induced RAW264.7 macrophages	Inhibiting the secretion of NO, IL-1*β*, IL-6 and TNF-*α* by p38MAPK and JAK-STAT1 signal pathway	Zhang et al. (2023)

8	*P. eryngii*	WPEP	Extraction with hot water at 70 °C, purification with DEAE-52 and Sephadex G-200	167/HPSEC	Xyl, Man, Glc, and Gal, with an approximatemolecular ratio of 21.35:3.28:73.22:1.63/GC	LPS-induced RAW264.7 macrophages	Inhibiting LPS-induced inflammation by regulating the production of NO, PGE-2, IL-1 *β*, TNF-*α* and IL-6, which was related to MAPK and NF- κB signal pathway.	Ma et al. (2020)
9		NPEP		274/HPSEC	Xyl, Man, Glc, and Gal, with an approximate molecular ratio of 1.13: 8.36: 84.04: 4.05/GC			
10	Cs-HK1 mycelial of *Cordyceps sinensis*	EPS	Fermentation, deproteinization	104/HPGPC	Glc, Man, Rib and Gal molecular ratio of5.349: 2.653: 0.755:1.000/PMP-HPLC	LPS-induced THP-1 and RAW264.7 cell, and LPS-induced acute intestinal injury	Inbibiting the release of NF-κB, NO, TNF-*α* and IL-1β *in vitro*, and suppressing the expression of TNF-*α*, IL-1*β*, IL-10 and iNOS and alleviating the intestinal injury.	Song et al. (2020)
11	*Craterellus cornucopioides*	CCPP-1	Extraction with hot water (90 °C) for 3 times, purification by DEAE-52 and Sepharose CL-4B	920/ELSD-HPLC	Man, Glc, Xyl, Ara, Fru in molar ratio of 0.7: 0.05: 0.18: 1: 0.05/HPLC	LPS-stimulated RAW264.7 macrophages	Inhibiting ROS and NO accumulation. decrease TNF-*α*, IL- 1*β*, IL-18 and iNOS expression by inhibiting NF-κB signaling pathway and NLRP3 inflammasome activation.	Xu et al. (2021a)
12	*Flammulina velutipes*	FVP<br>FFVP	Ultrahigh pressure-ultrasonic extraction	15961/HPLC<br>1.5702/HPLC	-<br>-	LPS-induced mice model	Reducing the secretion and mRNA expression of IL-1*β*, IL-6, IL-18, and TNF-*α*. The anti-inflammatory capacities were related to inhibiting the activation of an NLRP3 signaling pathway.	Ma et al. (2022)
13	Fruiting bodies of *Pleurotus citrinopileatus*	PCPS	Extraction with water, purification by DEAE-Sepharose chromatography and Sephacryl S-400 column	450.00	Glc/GC–MS	LPS/IFN-γ stimulated THP-1-derived macrophages, activated macrophages and human monocyte-derived macrophages	Inhibiting the secretion of pro-inflammatory cytokines and chemokines and promoting the expression of IL-10, its anti-inflammatory effect was related to Dectin-1 and TLR2 receptors	Minato et al. (2019)
14	Fruiting bodies of *Tremella fuciformis*	TFPS	Extraction with hot water (97.81°C for 6 h)	-	-	LPS-stimulated RAW264.7 macrophages	Inhibiting the inflammatory response by inhibiting the expression of miR155 and the activation of NF-κB	Ruan et al. (2018)
15	Sporoderm-broken spore *Ganoderma lucidum*	GLSP	Extraction by hot water at 100 ℃ for 2 h.	82/HPGPC	Glc (90.82%), Gal (7.95%) and Ara (1.23%)/GC–MS	LPS -induced EC-6 cells	Inhibiting the overproduction of NO, IL-6 and IL-1*β* to show anti-inflammatory activity	Wen et al. (2022)
16	Mycelium of *Hericium erinaceus*	EP-1	Extraction with water at 70 °C with gentle stirring for 12 h, purification with DEAE-Sephadex column	-	-	Acetic acid-induced ulcerative colitis rats	Improving the symptoms of colitis, regulating the intestinal microflora, increasing SCFAs, and inhibiting the expression of GPR41 and GPR43 in the colon of UC rats.	Shao et al. (2019)

17	P. eryngii	WP EP	--	167.00	Xyl, Man, Glc and Gal with a molecular ratio of 21.35: 3.28: 73.22: 1.63	DSS-induced colitis mouse model	Enrichmenting inhibition of immune cell proportion and pro-inflammatory cytokine overproduction, which were found to correlate with the key functional protein expression related to the NF-κB signaling pathway, and affect the imbalance of intestinal microflora.	Ma et al. (2021)
18	*Tremella fuciformis*	HTP s	Extraction with boiling water in a solid-liquid ratio of 1:20 (w/v) for 6 h, purification with DEAE-cellulose	288.6/HPGPC	Man, Rib, Rha, GlcA, Glc, Gal, Xyl, Ara and Fuc (9112057, 337.73, 623.67, 29067.09, 2086.89, 1656.21, 25035.97, 1540.07 and 21388.01 mg/kg)/LC	DSS-induced colitis mouse model	Reducing the proliferation of Foxp3+T cells and IgA-coated bacteria, enhancing the anti-inflammatory cytokines of colitis mice, reducing proinflammatory cytokines, and affecting the composition of intestinal flora and microbial metabolites.	Xu et al. (2021d)
19	*Scorias spongiosa*	SSPs	-	-	-	C57BL/6J mice	Enhancing the ability of anti-inflammation and anti-oxidation to contribute to intestinal health, but also enriching the diversity and composition of intestinal microflora.	Xu et al. (2022a)
20	*Lentinula edodes*	lenti nan	-	-	-	LPS induced inflammatory response in intestine of juvenile taimen	Improving the ability of intestinal anti-oxidation and anti-inflammation, and maintain a high level of Claudin d, κ, CAT and IκBα expression, effectively improving intestinal microflora and increasing the relative abundance of beneficial bacteria	Ren et al. (2019)
21	*Ganoderm a lucidum*	GLP	-	-	-	DSS-induced colitis in rats	Reducing disease activity index scores, increasiing SCFAs-producing bacteria (Ruminococcus_1) and reducing pathogens (Escherichia-Shigella) in the small intestine and cecum of rats, producing more SCFAs, regulating11 genes rich in 6 inflammatory pathways, enhancing immunity, and reducing inflammatory reaction	Xie et al. (2019)
22	*Flammulin a velutipes*	FVP	Extraction with water at 80°C.	7473.14 (48.09%) and 15.077 (51.91%)/HPGFC	Man, Glc, Xyl, Ara and Fuc with the molar ratio of 6.6:27.8:18:1.5:5.2/ GC	DSS–induced ulcerative colitis in mice	Regulating the relative mRNA expression of cytokines and tight junction proteins, promoting the production of single-chain fatty acids, and regulating intestinal microorganisms.	Zhao et al. (2020)
23	*Flammulin a velutipes*	FVP	Boiling with distilled water for 2 hours	-	-	DSS-induced colitis in rats	Regulating the intestinal microbial metabolism, increasing the level of SCFAs in cecum, down-regulating TLR4/NF-κB signal pathway, and improving colitis.	Zhang et al. (2020)
24	*Flammulin a velutipes*	FVP	Extraction with water at 91℃ for 2h, purification with macroporous resin	66.3793, 2.4670/GPC	Man, Rib, Glc, Gal, Xyl, Ara and Fuc, and GlcA with mole ratio of 7.06: 0.59: 72.02: 14.15: 1.39: 0.06: 4.15: 0.59./HPLC	Cd-induced gut injury in mice	Reducing intestinal inflammation and barrier disruption, modulating the gut microbiota and restoring SCFAs levels, improving gut microbiota's metabolic functions and SCFAs-mediated energy metabolism.	Hao et al. (2023)

25	Fruiting bodies of *Lentinula edodes*	—	Extraction with distilled water at 100 °C for 6 h, purification with DEAE-sepharose CL-6B	-	-	Caco-2 cells; DSS-induced colitis in mice	Inhibiting the necrotizing cell death of colitis and Caco-2 cells in mice, inhibiting the signal cascade of RIPK1-RIPK3-MLKL necrotizing ptosis, and reducing the level of p-MLKL in the colon of colitis mice.	Alagbaos and Mizuno (2021)
26	Fruiting bodies of Lentinula edodes		Extraction with distilled water at 100°C for 6 h, purification with DEAE-sepharose CL-6B	-	-	DSS-induced colitis in mice	Inhibiting the expression of TNF-*α*, IL-6, IL-1 *β* and IFN-*γ*, and inhibiting colitis in mice.	Alagbaos and Mizuno (2022)
27	*Morchella importuna*	MIPs	-	35.54/HPGPC	28 700.0–34 000.0, 34 000.0–40 000.0, 40 000.0–43 000.0, 43 000.0–47 738.0 with molar ratios of 47.1: 30.4: 12.5: 10.0/Ion-exchange chromatography	CCl_4-induced hepatic damage in mice	Promoting antioxidant and anti-inflammatory activities to injury	Xu et al. (2021c)
28	Fruiting bodies of *Flammulina velutipes*	FVPs	Extraction with hot water (100°C for 6 h), purification by diethylaminoethyl cellulose	2779371g/mol/HPGPC	Man (68796.89 mg/kg), Rib (539.35 mg/kg), Rha (243.35 mg/kg), GlcA (50.64 mg/kg), GalA (96.24 mg/kg), Glc (54900.80 mg/kg), Gal (88339.75 mg/kg), Xyl (22228.41 mg/kg), and Fuc (30248.37 mg/kg)/LC	CCl_4-induced hepatic damage in mice	Attenuating hepatic injury by promoting antioxidant and anti-inflammatory effects. Changing the composition of the gut microbiome and regulating certain bacterial pathways associated with fatty acid biosynthesis, tryptophan metabolism, and metabolism of xenobiotics by cytochrome P450 to protect the liver from the toxic effects of CCl_4.	Xu et al. (2022b)
29	*Pleurotus ostreatus*	POP	Extraction with distilled water by using an ultrasonic-assisted cellulose method, purification using DEAE-2 and Superdex 200	-	-	CCl_4-induced ALI	Exhibiting hepatoprotective effects, and the mechanism is correlated with antioxidants that regulate metabolic pathway disorders and alleviate liver mitochondria apoptosis.	Zhu et al. (2019)
30	*Pleurotus ostreatus*	POP<br>PPOP	Extraction by hot water, and purification by DEAE-52 and Sephadex G-100 chromatography	24/HPSEC<br>17, 74, 81/HPSEC	HPLC<br>Rha, GalA and Xyl in a molar ratio of 0.10:1.98:1.00/HPLC	CCl4-induced liver injury in mice	Scavenging free radicals, preventing lipid peroxidation and improving endogenous antioxidant defense system	Duan et al. (2020)
31	Fruiting bodies of *Ganoderma lucidum*	GLPS	Boiling water extraction with alcohol precipitation	-	-	CCl_4-induced liver injury in mice	Inhibiting the activation of NLRP3, reducing inflammation and inhibiting free radical lipid peroxidation.	Chen et al. (2019)

32	Strain of *Pleurotus geesteranus*	EPS	Fermentation, extraction, and purifition by DEAE-52 and Sephadex G-100 column	-	Ara, Gal, Glc, Man, Rha and Xyl with molar ration of 5.38: 2.70: 200.34: 8.93: 9.3: 4.20/GC	Alcohol-induced liver injury	Decreasing the levels of total cholesterol, triglyceride, cytochrome P4502 and inflammatory mediators (TNF-*α*, IL-6, IL-1, COX-2, NO and iNOS)，increasing the activities of SOD, GSH-Px and CAT, and reducing the state of lipid peroxidation.	Song et al. (2018)
33	Mycelium of *Coriolus versicolor*	PSP-1b1	-	21.70	Fuc, Gal, Xyl, Man, GlcA and Glc at a relative molar ratio of 0.16: 0.60: 0.02: 0.55: 0.04: 1.00.	Alcohol-induced liver injury	Decreasing the levels of ALT and AST, up-regulating the expression of SOD and CAT, down-regulating the expression of Cyp2E1, iNOS, and HO-1, and decreasing the expression of TLR4, MYD88, CD14, IL-1*β* and TNF-*α*.	Wang et al. (2019)
34	*Morchella esculenta*	MEPs	Extraction by distilled water in a 90℃ for 2 h, and purifition by DEAE-52 cellulose column	504.274 (15.739%), 48.229 (19.984), 19.714 (38.874), 13.115 (18.435) and 6.473 (6.967)/HPGPC	GlcN, Gal, Glc and Man in the molar ratio of 0.009: 0.053: 0.900: 0.037/IC	DSS-induced liver injury in mice with acute colitis	Increasing SOD, CAT, and GSH-Px activities and decreasing MDA and MPO levels	Chen et al. (2023b)
35	Fruiting bodies of *Grifola frondosa*	GFP	Extraction three times by water at 80℃ for 2 h, purifition by DEAE-Sephadex A-25 and Sephadex G-100	155/HPLC	Rha, Xyl, Man and Glc with molar ratio of 1.00: 1.04: 1.11: 6.21/GC	LPS/D-galactosamine-induced acute liver injury	Inhibiting the activities of ALT and AST, MPO and the levels of the TNF-α, IL-2, IL-6, and MCP-1, and MDA content. Upregulating SOD, GSH, transcription factors Nrf2, Nqo-1, and HO-1 and downregulating transcription factor Keap-1 in the Nrf2/ARE signaling pathway.	Meng et al. (2021)
36	*Coprinus comatus*	CCP	Extraction by water (1:30) at 90 °C for 3 times, deproteinization by Sevage reagent	3740/HPGPC	Glc (26.36%), Gal (15.21%), Man (0.75%), Rha (0.57%) and GlcA (0.55%)/HPLC	Alcohol-induced liver injury in mice	Reducing the levels of AST, ALT and TG in serum, promoting the proliferation of beneficial bacteria and inhibiting the proliferation of harmful bacteria.	Yu et al. (2024)
37	*Ganoderma lucidum*	GLP	Extraction at 80℃ for 2 h	-	Glc, Man, Rib and GlcA in the content ratio of 44: 11: 1:1/HPLC	LPS-induced acute pneumonia	Inhibiting the infiltration of inflammatory cells, reducing the release of GM-CSF, IL-6 and the gene expression of IL-1*β*, IL-6, TNF-*α* and Saa3, inhibiting the activation of Nrp1, up-regulating the levels of Bcl2/Bax and LC3, and down-regulating the expression of C-Caspase 3/Caspase3 and p62.	Zhang et al. (2022b)
38	Residue of *Lentinula edodes*	RPS, ARPS and ERPS	Water-extraction (80 ℃, 3 h), alcohol-precipitation (1:3, v/v, 4 ℃, 12 h), and then centrifugation (10,000 ×g, 10 min) to gain RPS；enzymatic-hydrolysis with nailase to get ERPS; acidic-hydrolysis	-	RPS: Rha (12.96%), Ara (27.36%), Man (3.64%), Gal (9.81%) and Glu (46.23%), ARPS: Rha (32.4%), Ara (22.53%), Gal (8.30%) and Glu (36.70%), ERPS: Rha (81/97%), Ara (4.63%), Man (2.93%), Gal (4.75%) and Glu (5.72%), respectively/GC	LPS-induced lung injured mice	Ameliorating the lung W/D ratio, reducing the TNF-*α*, IL-6, and IL-1*β* levels, lowing the pulmonary MPO activity, decreasing the serum C3 and hs-CPR contents, as well as improving the antioxidant status by enhancing pulmonary enzyme activities (SOD, GSH-Px, CAT, and T-AOC) and eliminating the lipid peroxidation (MDA and LPO), respectively.	Ren et al. (2018)

			with H_2SO_4 to get ARPS					
39	Fruiting bodies of *Lentinus edodes*	PLE	_	-	GlcA, Man, Gal, and Glc with a molar ratio of 0.35:0.33:0.49:17.63/HPLC	Zymosan (0.5 mg/g bw)-induced acute lung injury	Reducing the levels of serum lung injury indicators (C3, hs-CRP, and GGT), reducing the levels of inflammatory factors (TNF-α, IL-1β, and IL-6), and increasing the activities of antioxidant enzymes (SOD and CAT) in the lung.	Zhang et al. (2022c)
40	*Morchella esculenta*	SFMP-1	Sulfated derivative	7.0/HPLC-ELSD	-	$PM_{2.5}$-induced NR8383 cells inflammation	Reducing $PM_{2.5}$-induced cell death, cell apoptosis and production of TNF-α and IL-1β, and ROS formation. Down-regulating the expression of iNOS and COX-2. The mechanism was related to inhibite NF-κB activation.	Li et al. (2019)
41		CFMP-1	Carboxymethylated derivative	6.9//HPLC-ELSD	-		Show weak effects	
42	Fruiting bodies of *Trametes orientalis*	TOP-2	Ultrasonic-microwave assisted extraction, DE-52 ion-exchange cellulose and Sephadex G-100 chromatography	-	-	$PM_{2.5}$-induced lung injury in mice.	Alleviating pulmonary edema, attenuating the toxic effects, inhibiting the activation and migration of neutrophils, alleviating the $PM_{2.5}$-induced damage on lung parenchyma cells, maintain the cell membrane per-meability, inhibiting the release of pro-inflammatory cytokines including TNF-*α*, IL-1*β* and IL-6, increasing the activities of SOD, CAT and GSH-Px, reducing the levels of MDA, PCG and 8-OHdG, up-regulating the expressions of Nrf2 and HO-1, and inhibiting the activation of the NLRP3 inflammasome.	Zheng et al. (2019)
43	*Cordyceps cicadae*	CPA-1 and CPB-2	-	-	-	High fructose/high fat diet induced obesity and metabolic disorders in rats.	Showing protective effects on insulin resistance, metabolic abnormalities, liver oxidative stress and inflammation.	Zhang et al. (2021)
44	Fruiting bodies of *Grifola frondosa*	GFPA	Extraction twice with hot water at 80°C for 2 h, purifition using a DEAE-52 anion exchange column , HiPrep™ 26/60 Sephacryl™ S-400 high-resolution and EzLoad 26/60 Chromdex 200	5570.17/gel chromatography-differential-MALLS	Glc, Gal and Fuc at a molar ratio of 99.73: 0.17: 0.10/HPAEC	HFD-fed mice	Reducing liver steatosis and inflammation, showing a strong weight loss effect. These effects are related to the inhibition of chronic inflammation by TLR4/NF-kB signal transduction pathway.	Jiang et al. (2022)
45	*Ganoderma lucidum*	BSGLP	Oscillated extraction (300 rpm) at 70 ℃ for 12 h	26.0/HPGPC	Glc, Man, and Gal in a molar ratio of 87.4: 4.81: 8.14/GC	HFD-induced obesity in mice	Reducing fat accumulation, liver steatosis, inflammation and hyperlipidemia. Its weight loss effect may be related to the regulation of intestinal microflora, intestinal barrier, the production of single chain fatty acids, the activation of GPR43 and the inhibition of TLR4/MYD88/NF- κB signal pathway.	Sang et al. (2021)

46	Fruiting bodies of *Agrocybe cylindracea*	ACP	Extraction 3 times with distilled water (1:30, w/v) at 85℃ for 3.5 h,	670/GPC	Man, Rib, Rha, GlcA, Glc, Gal, and Xyl in a molar ratio of 0.29: 0.47: 0.25: 1.72: 1.51: 0.94:1 /HPLC-PDA	High-fat diet (HFD)-induced obese in mice	Improving diet-induced obesity and related metabolic diseases, inhibiting the secretion of pro-inflammatory factors, and regulating intestinal microorganisms and related metabolites.	Zhu et al. (2022)
47	Spent mushroom compost of *Lentinula edodes*	ASMCP	Water-extraction (85 oC, 3 h), alcohol-precipitation (1:3, v/v, 4℃, 12 h), H_2SO_4-hydrolysis, and purification with DEAE-cellulose and Sephadex G-100 column	2.177/HPGPC	Rha (32.36%), Ara (22.58%), Gal (8.92%) and Glc (36.14%), with molar ratios of 4:3:1:4/GC	LPS-induced kidney injury	Decreasing the levels of serum TNF- α, IL-6 and IL-1β, inhibiting lipid peroxidation, decreasing the levels of BUN, CRE and UA, improving antioxidation by increasing the activities of SOD, GSH-Px, CAT and T-AOC.	Song et al. (2020)
48	Fruiting bodies of *Cordyceps cicadae*	CCP	Extraction with distilled water (1:10, w/v) three times at 90 °C for 6 h.	-	Man, Rha, Glc, and Gal with molar ratios of 12.71: 1.53: 1.0: 12.64/HPLC	High-fat diet, STZ-induced Diabetic nephropathy in rats	Decreasing the levels of TNF- α, IL-1 β, IL-6 and CTGF, MMP2, decreasing the content of HYP, increasing the relative abundance of relative abundance of Lactobacillus, Bacteroides and Akkermansia, and significantly increasing the relative abundance of Desulfovibrionaceae, Ruminococcus, Prevotellaceae, Paraprevotella and Oscillospira.	Yang et al. (2020)
49	Fruiting bodies of *Floccularia luteovirens*	FLPs	Extraction twice with hot water at 90℃ for 5 h	-	-	Diabetic nephropathy	Improving and reducing the renal tissue damage caused by high glucose, targeting and regulating phosphorylated GSK-3β, and inhibiting the accumulation of inflammatory factors. Activation of Nrf2/HO-1 pathway and enhancement of CAT activity.	Wang et al. (2023)

Note: HPLC: High performance liquid chromatography; HPSEC: High performance size exclusion chromatography; HPGFC: High performance gel filtration chromatography; SEC-RI-MALLS: Size exclusion chromatography-refractive index multi-angle laser light scattering; SEC-MALLS-VIS-RID: Size exclusion chromatography coupled with multi-angle laser light scattering, viscometry and refractive index detector; RPLC-QQQ-MS: Reversed phase liquid chromatography-tandem quadrupole mass spectrometry; GC-MS: Gas shromatography-mass spectrometer HPAEC: High performance anion exchange chromatography; HPGPC: High performance gel permeation chromatography; LC: Liquid chromatography; GPC: Gel permeation chromatography; IC: Ion chromatograph HPLC-ELSD: High performance liquid chromatography-evaporative light scattering detector; HPLC-PDA: High performance liquid chromatography-diode matrix detector; DEAE-52: Diethylaminoethyl cellulose-52: Fuc: Fucose; Gal: Galactose; Xyl: Xylose; Man: Mannose; Glc: Glucose; Ara: Arabinose; Rib: Ribose; GlcA: Glucuronic acid; Fru: Fructose; Rha: Rhamnose; Fuc: Fucose; GalA: Galacturonic acid; GlcN: Glucosamine
